# Universal Method of Compatibility Assessment for Novel Ablation Technologies With Different 3D Navigation Systems

**DOI:** 10.3389/fcvm.2022.917218

**Published:** 2022-06-28

**Authors:** Luigi Pannone, Ivan Eltsov, Robbert Ramak, David Cabrita, Marc Verherstraeten, Anaïs Gauthey, Antonio Sorgente, Cinzia Monaco, Ingrid Overeinder, Gezim Bala, Alexandre Almorad, Erwin Ströker, Juan Sieira, Pedro Brugada, Mark La Meir, Gian-Battista Chierchia, Carlo de Asmundis

**Affiliations:** ^1^Heart Rhythm Management Centre, Postgraduate Program in Cardiac Electrophysiology and Pacing, Universitair Ziekenhuis Brussel - Vrije Universiteit Brussel, European Reference Networks Guard-Heart, Brussels, Belgium; ^2^Medtronic Inc, Minneapolis, MN, United States; ^3^Boston Scientific, Marlborough, MA, United States; ^4^Department of Cardiac Surgery, Universitair Ziekenhuis Brussel - Vrije Universiteit Brussel, Brussels, Belgium

**Keywords:** catheter ablation, universal compatibility, DiamondTemp ablation system, Rhythmia electroanatomic mapping system, cardiac arrhythmias

## Abstract

**Background:**

New technologies for ablation procedures are often produced by different companies with no cross-compatibility out of the box. This is not a negligible clinical problem since those separately developed devices are often used together. The aim of this study was to develop a bench-testing method to assess compatibility between the DiamondTemp ablation system (DTA) and the Rhythmia electroanatomic mapping system (EAM).

**Methods:**

Different setups were tested. DTA was connected to the Rhythmia EAM using the following configurations: 3.1. An Ensite EPT GenConnect box (GCB) and Rhythmia Maestro GCB (Maestro GCB, native Rhythmia setup); 3.2. The Medtronic GCB-E and Maestro GCB; 3.3. The Medtronic GCB-E out via the Medtronic GCB-E directly to the Rhythmia at box 1 (pin A61 to A64).

**Results:**

The DTA location was represented in real-time on the Rhythmia EAM. A proper tracking of the DTA was observed in all setups tested by visual comparison of physical catheter movements and its representation on EAM. In configuration 3.1, a significant shift was observed after the first radio frequency (RF) application; however, further applications caused no further shift. In setup 3.2, no significant shift was observed. The setup 3.3 showed a massive shift in the catheter position before ablation compared to baseline points acquired using the Orion catheter as a reference.

**Conclusions:**

A universal and reproducible solution for compatibility testing between the various mapping systems and the ablation catheters has been described. DTA has been demonstrated as compatible with Rhythmia EAM with satisfactory results if a specific setup is used.

## Introduction

The medical device industry is a fast-growing field and various companies are developing novel products to ensure precise and personalized treatment for cardiac arrhythmias.

New technologies are often produced by different companies with no cross-compatibility out of the box. This is not a negligible clinical problem since those separately developed devices are often used together. In the absence of a compatibility statement from the companies involved, their use may be considered “off label”.

One of the most challenging clinical settings is the use of an ablation catheter with a 3D-electroanatomic mapping system (EAM), when both systems are produced by different, often competitive, manufacturers. These systems are intended to create 3D models of the dedicated heart chamber and to track the ablation catheters in the model with sufficient precision to deliver therapy.

All existing EAMs use 2 main catheter tracking technologies: magnetic field-based or impedance-based technology, either separately or in a hybrid mode (1).

The magnetic-based systems are using a magnetic field generator placed under the patient's table, which creates a magnetic field around the patient's heart. Catheters introduced into the patient have a magnetic sensor, constantly sending localization information based on the magnetic field measurements, which allows the system to track it and represent it in the 3D model. In this case, the catheter is developed by the manufacturer of the EAM, ensuring compatibility.

Impedance-based tracking allows visualizing and tracking virtually any catheter using the impedance measurement between the external patches and an electrode on the catheter. However, the precision of the tracking system may be influenced by external factors such as locator signal frequencies, system tracking algorithms, catheter electrode spacing, filter settings, cables, interface cables, and RF energy delivery. The hybrid tracking systems are using both the magnetic and impedance-based methods where magnetic tracking enhances localization accuracy and impedance measurement allows visualizing the third party catheters. The Rhythmia mapping system (Boston Scientific MA) is one of the most common EAMs, which uses a hybrid tracking algorithm.

The novel DiamondTemp^TM^ ablation system (DTA), (Medtronic Inc, Minneapolis, MN) and Rhythmia^TM^ EAM (Boston Scientific) are both CE-Marked and FDA-approved medical devices. The DTA has been validated by its manufacturer only in combination with Ensite Precision^TM^ System (Abbott, Inc., Chicago, Illinois) (2).

The aim of this study was to develop a bench testing method to ensure the compatibility between the DTA and Rhythmia EAM.

## Methods

### Ablation Catheter and Mapping System

The DTA ablation catheter is a 7.5-F irrigated radiofrequency (RF) catheter; the 4.1-mm composite tip electrode delivers RF. The ablation electrode tip is embedded with 2 interconnected diamonds which allow rapid RF delivery (due to isoelectric diamond properties) and shunting heath from externalized thermocouples. This allows accurate temperature measurement at the tip-tissue interface. The catheter operates in a temperature control mode and a dedicated RF generator (RFG) titrates rapidly the delivered power to the target temperature. The dual composite ablation tip behaves as a single electrode during ablation and the electrical insulation of the tip allows for high-resolution EGM sensing (3, 4).

Rhythmia^TM^ is a high-density EAM that offers detailed insights into tissue electrical activity (5). Indeed, the reliability of electrogram annotation, the density of recorded electrograms, the low noise, and the enhanced characteristics of recording make Rhythmia^TM^ a reliable tool in clinical practice (6).

### Configurations for Compatibility Assessment

The following endpoints were evaluated for compatibility assessment: (1) there is no energy leak or a sudden shortcut within the proposed connectivity configuration, which ensures that the RF energy power and time shown on the device correspond to the delivered power and therapy duration, and (2) the DTA is correctly represented inside the 3D model, with a precise and reliable position not influenced by external factors, especially RF energy delivery. Four different configurations were tested.

The DTA can be connected to the navigation system either by using EGM out cables from the RF generator or by using special ablation connection boxes (provided by the mapping system manufacturer) to filter out RF energy so that it does not affect localization. DTA is using Maestro-type connectors and all the following experimental configurations are based on that.

**Figure 1:**
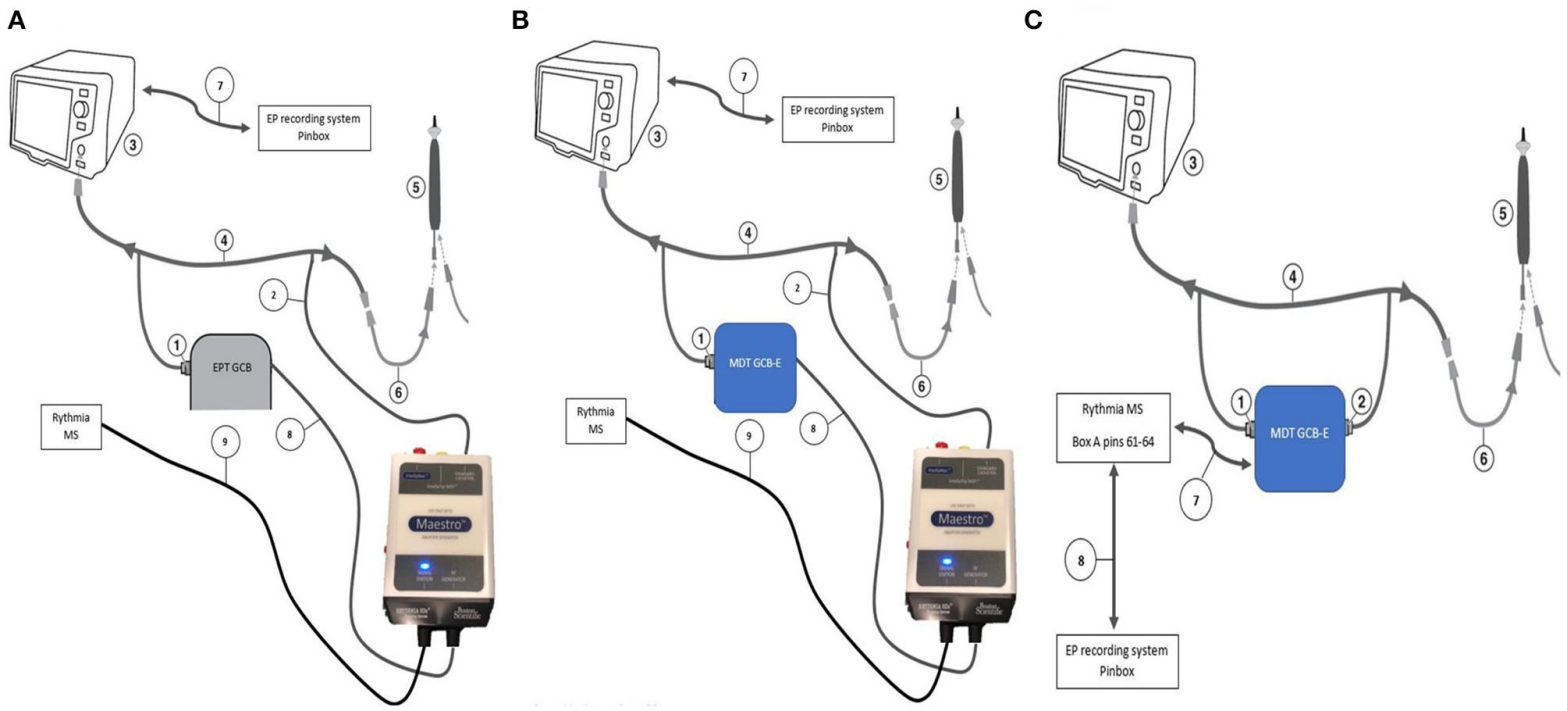
DiamondTemp and Rhythmia connectivity configurations. **(A)** Setup 3.1. An EPT GCB and Rhythmia Maestro GCB (Maestro GCB, native Rhythmia setup). (1) 14-pin twist connector to RFG via the GCC; (2) 9-pin quick connector to DTC via the GCC to the DTC to RFG cable; (3) DTA RFG; (4) DTA GCC; (5) DTC; (6) DTC to RFG cable; (7) IC out cable; (8) 9-pin quick connector of the Maestro GCB; and (9) Connector from the Maestro GCB to the Rhythmia MS. **(B)** Setup 3.2. This configuration shows the best accuracy results—The catheter connection is connected to Rhythmia GCB using Genconnect cable and then to MDT GCB as shown in the picture. (1) 14-pin twist connector to RFG via the GCC; (2) 9-pin quick connector to DTC via the GCC to the DTC to RFG cable; (3) DTA RFG; (4) DTA GCC; (5) DTC; (6) DTC to RFG cable; (7) IC out cable; (8) 9-pin quick connector of the Maestro GCB; and (9) Connector from the Maestro GCB to the Rhythmia MS. **(C)** Setup 3.3. The MDT GCB-E and IC out via the MDT GCB-E directly to the Rhythmia IC in at box 1 (pin A61 to A64) (1) 14-pin twist connector to RFG via the GCC; (2) 9-pin quick connector to DTC via the GCC to the DTC to RFG cable; (3) DTA RFG; (4) DTA GCC; (5) DTC; (6) DTC to RFG cable; (7) IC out cable; and (8)Rhythmia IC out to the EP recording system.

Testing configurations:

1.0 DTA connected only to the DTA RFG - No GenConnect (GC) nor Genconnect Cable (GCC) connected to the DTA
  1.1 An Ensite EPT GenConnect box (EPT GCB).
  1.2 The Medtronic (MDT) Generator Connection Box E (MDT GCB-E).
  1.3 The MDT GCB-E and intracardiac (IC) signal out via the MDT GCB-E.
2.0 DTA not connected to a mapping system (MS) but connected to
  2.1 An Ensite EPT GenConnect box (EPT GCB).
  2.2 The Medtronic Generator Connection Box E (MDT GCB-E).
  2.3 The MDT GCB-E and IC out via the MDT GCB-E.
3.0 DTA connected to the Rhythmia MS using (Figure 1)
  3.1 An EPT GCB and Rhythmia Maestro GCB (Maestro GCB, native Rhythmia setup).
  3.2 The MDT GCB-E and Maestro GCB.
  3.3 The MDT GCB-E and IC out via the MDT GCB-E directly to the Rhythmia IC in at box 1 (pin A61 to A64).

Configurations 1.x and 2.x were used for functional and safety assessment only.

### Functional and Safety Parameters Assessment

To assess the functional and safety parameters of the DTA with the different setups proposed, a calibrated electrosurgery analyzer “FLUKE Biomedical QA-ESII” was used. This device allows for the continuous measurement of power, current, peak-to-peak voltage (closed load only), and crest factor for each RF application. (7) The test and connectivity of the FLUKE Biomedical QA-ESII Electrosurgery Analyzer equipment to different components is performed in “continuous operation mode” with no footswitch. In this mode, the analyzer continues to perform measurements continuously. The test is interrupted by pressing the “stop” key. The analyzer acts like a meter during the test, showing increasing and decreasing values as received from the unit being tested, in this case, the DTA RFG. The DTA RFG is connected to the connection for the electrode outputs of internal variable resistance. An active connection (Red) is directly connected to the catheter tip alligator clip wired to the Red pin. The neutral connection is directly wired from the DTA neutral plate connection and the analyzer neutral pin (Black).

The functional and safety parameters of the DTA and its RFG were assessed at 3 different loads, namely 50 Ohms, 100 Ohms, and 150 Ohms, and 3 different maximum power outputs, namely 50 W, 30 W, and 15 Watts. For each load and maximum power output, 3 measurements were performed to assess the following parameters: (1) maximum power output indicated on the DTA RFG; (2) power output measured at the tip of the DiamondTemp catheter (DTC); (3) current measured at the tip of the DTC; (4) peak-to-peak voltage measured at the tip of the DTC; and (5) maximum current variation among the 3 measurements.

### Assessing the DTA Reliability With EAM

**Figure 2:**
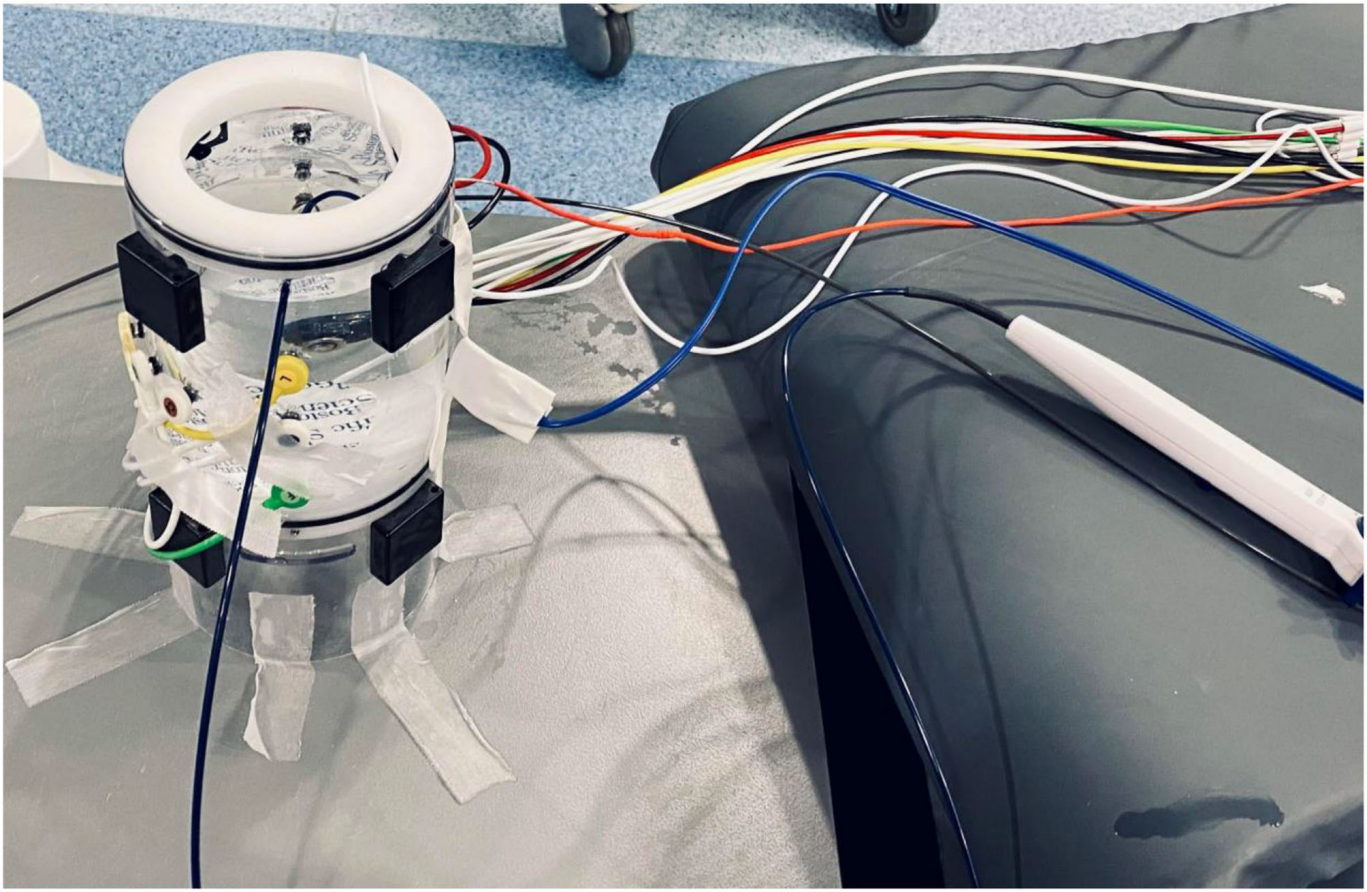
Accuracy testing setup “*in vitro*”. Dedicated testing phantom with all EAM cables connected, IntellaMap Orion catheter, and DiamondTemp ablation catheter submerged in the saline within the phantom.

The DTA reliability when using the Rhythmia^TM^ EAM was assessed as per the manufacturer's accuracy by testing work instruction using a dedicated EAM phantom. This ensured an accurate assessment of the EAM reliability using the DTA as this work instruction is normally used during the EAM installation prior to the certification for clinical use. Different components were connected to the DTA RFG and EAM according to the different setups to be tested (Figure 2).

**Figure 3:**
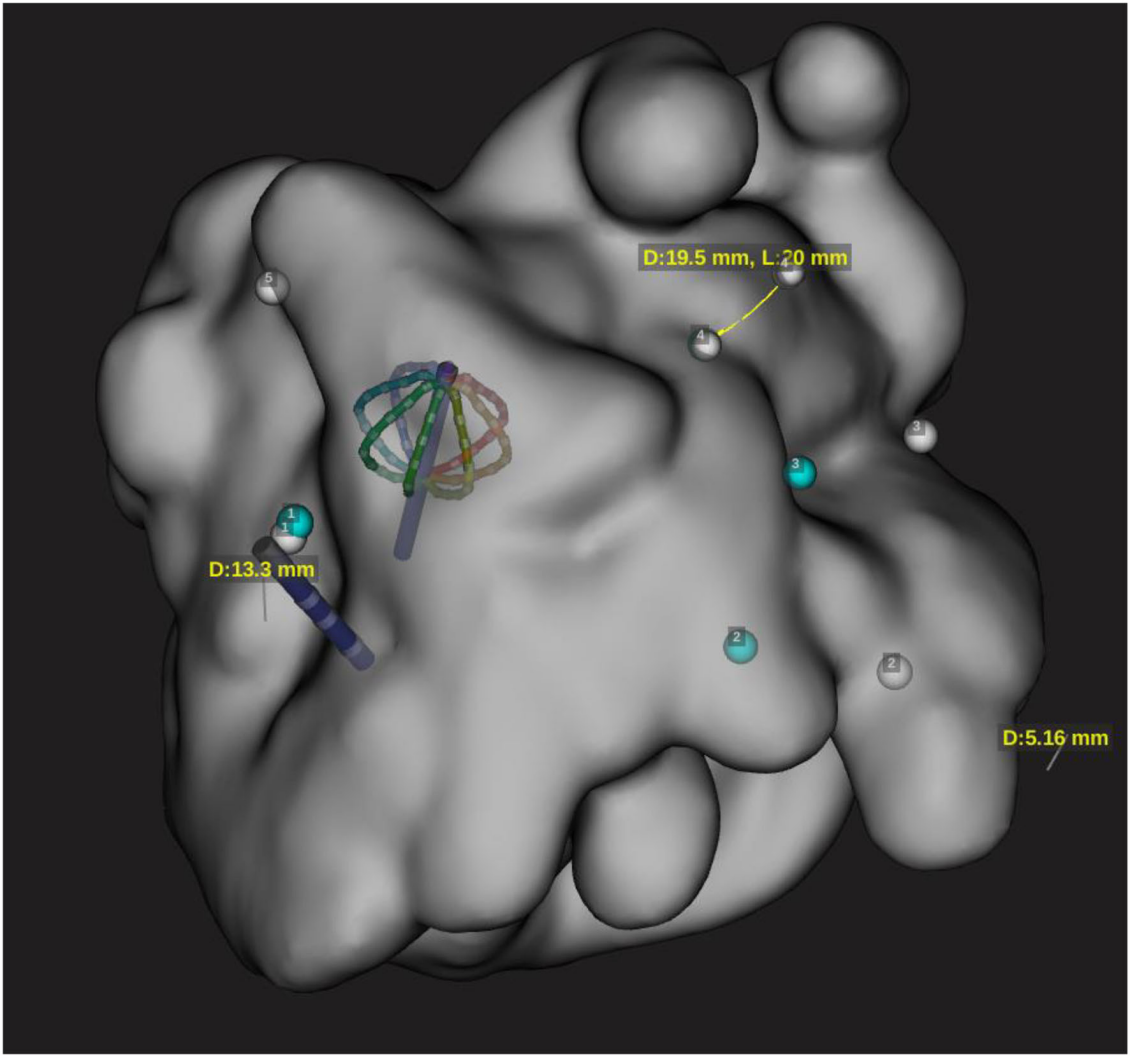
Representation of both Orion and DT catheter in the 3D map on the EAM. This snapshot is showing catheter shift during RF application with configuration 3.1 and distance measurement of 19 mm for configuration 3.3.

**Figure 4:**
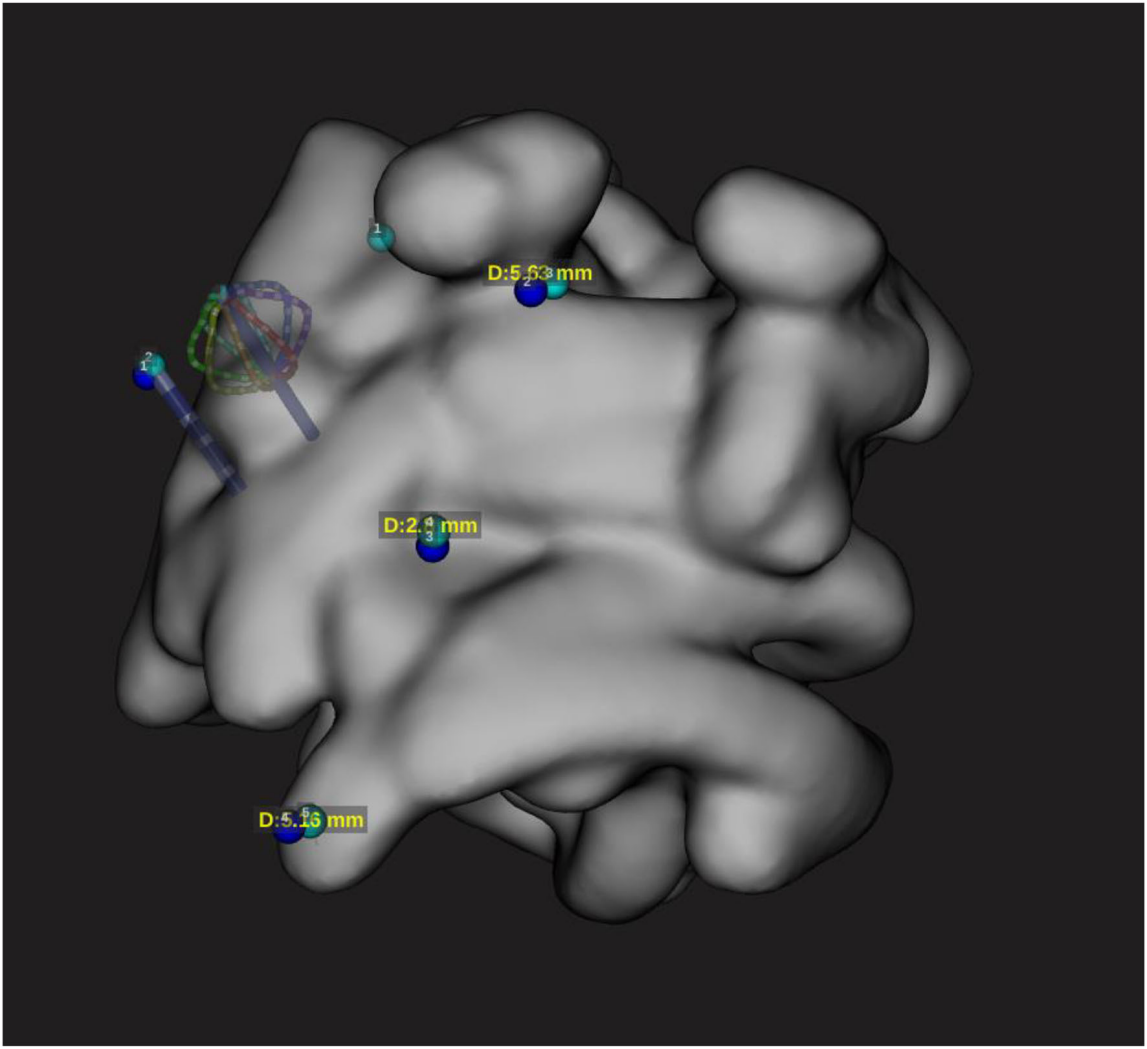
Localization accuracy with minimal deviation according to configuration 3.2. The catheter positioned on the reference points is represented on the EAM with minimal difference to the reference sensor-enabled catheter.

**Figure 5:**
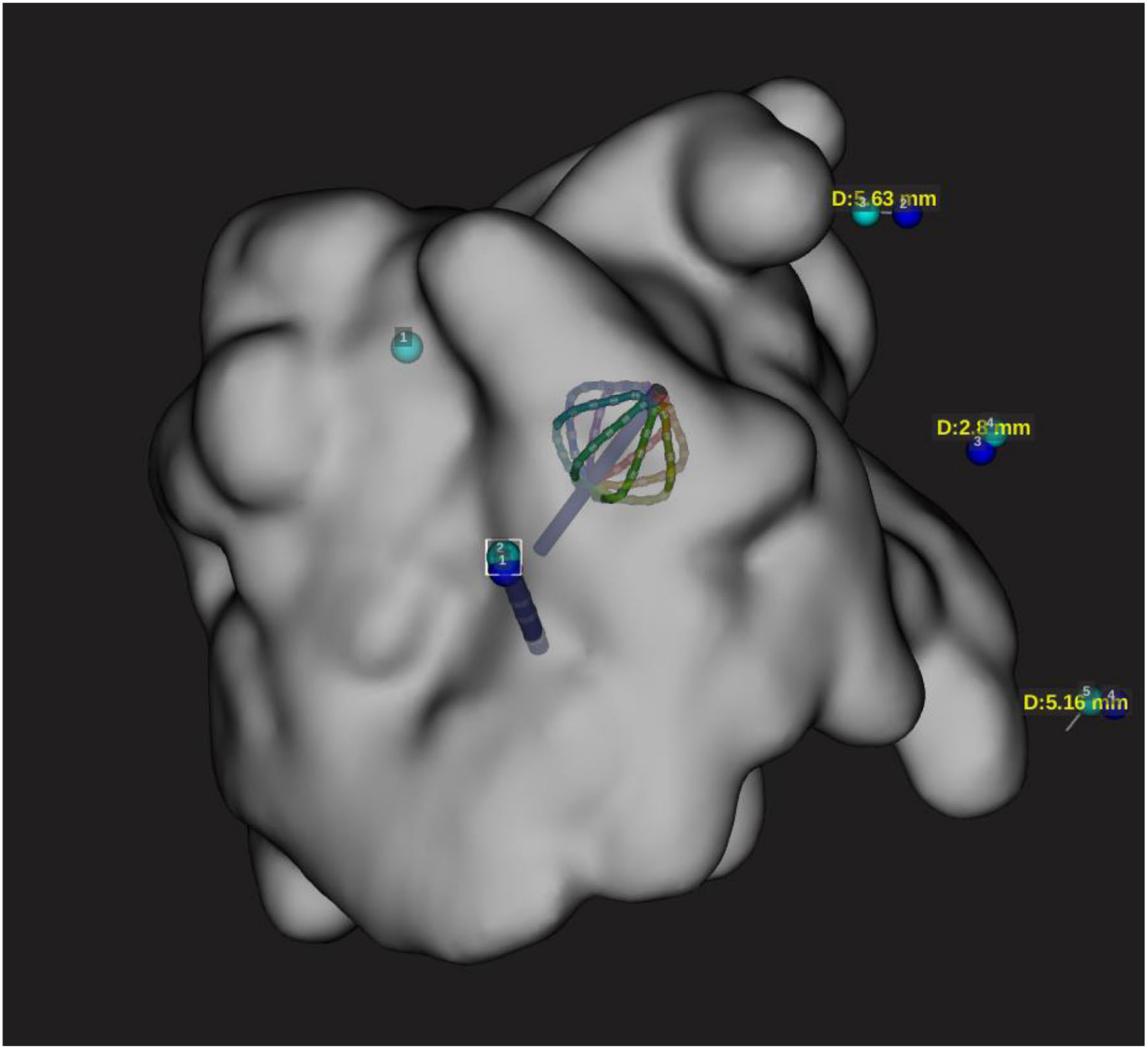
Configuration 3.2 during RF delivery. Localization accuracy of DTA catheter on the EAM during RF application (50w 30 s). This configuration has shown no catheter shift during RF application.

The phantom anatomy was built using a dedicated high-resolution mapping catheter, Intellamap Orion (Boston Scientific). Reference locations from the phantom were reached with the tip of this catheter and the reference locations were added to the surface of the phantom as a baseline. Then the tracking of the DTA was verified by visual comparison of the physical catheter movements and its representation on the map (Figures 3–**5**).

Before testing any additional variables, points were collected on the EAM with the DTA manually positioned at the established reference locations. These were the baseline points for each setup that was tested.

To assess the reliability of the catheter location on the EAM, the DTA was placed back at each reference location and an additional point was collected. An assessment was made regarding the reproducibility of the baseline point locations by measuring the distance between tags (in mm) on the EAM.

For each setup, three different RF pulses were tested: (1) RF energy for 45 s with a target temperature of 60°C (longest application time and highest temperature allowed by the DTA in a clinical setting), with 0 s of power ramp delay, 1 s of pre-cooling, and 0 s of cooling post ablation (RF1); (2) RF energy for 10 s with a target temperature of 55°C (longest application time and highest temperature allowed by the DTA in a clinical setting) with 0 s of power ramp delay, 1 s of pre-cooling, and 0 s of cooling post ablation (RF2); or (3) RF energy for 5 s with a target temperature of 50°C (longest application time and highest temperature allowed by the DTA in a clinical setting) with 0 s of power ramp delay, 1 s of pre-cooling, and 0 s of cooling post ablation (RF3).

Between each RF delivery, the DTA was removed and reinserted in the phantom and the DTA cable was disconnected and reconnected without moving the catheter.

After each RF application, the catheter was manually placed at each reference location by looking at the phantom directly. An additional point was collected at each reference location. Distance between every taken point and its corresponding baseline marker were measured using the EAM software.

Distance measurements (in mm) between baseline points and collected points were repeated for every reference location on the phantom model after each RF pulse. At the next step, the DTA was placed once again back at each reference location and an additional final point was collected.

### Configurations and Connections of the DTA to the Rhythmia EAM

The original Rhythmia^TM^ ablation setup has been modified to use DTA RFG by connecting DTA GCC and one of GCB to Maestro connections of the Maestro GCB. The reliability and accuracy of the DTA visualization on the Rhythmia^TM^ EAM were based on the fact that the Rhythmia^TM^ EAM has a hybrid-tracking mechanism. This hybrid mechanism uses an algorithm that calculates impedance values, from each electrode of nonmagnetic catheters, in the magnetic field map, created using the Orion catheter. This allows to accurately track nonmagnetic sensor-enabled catheters (6). The test was performed using the dedicated Boston Scientific Wet/Dry Tank corresponding to the Boston Scientific Work Instruction and accordingly to the EAM IFU (8).

A new ablation catheter has been configured in the EAM.

The anatomy shell and a Field Map were created using the Orion catheter as per IFU (1) to allow impedance tracking for 3rd party catheters. The DTA was connected to the Rhythmia^TM^ EAM according to setup 3.1. Five fixed metallic points inside the wet tank were used as references, with points 3, 4, and 5 being the closest to the X-ray flat detector. To test the influence of magnetic distortion, the X-ray flat detector was kept in sufficient proximity to the phantom. Points 1 and 2 were not affected by the magnetic field distortion while points 3, 4, and 5 were, with 5 being the most affected location by magnetic distortion. All 5 points were found within the range of the Rhythmia^TM^ EAM specifications. The Orion catheter was used to ensure the precision of all baseline points.

After preparation, the accuracy checks have been performed as described earlier.

### Statistical Analysis

All variables were tested for normality with the Shapiro–Wilk test. Normally, the distributed variables were described as mean ± standard deviation and the groups were compared through ANOVA, paired *t*-test, or unpaired *t*-test as appropriate, while the non-normally distributed variables were described as median (Inter Quartile Range) and compared using the Kruskal–Wallis test, the Mann–Whitney test, or the Wilcoxon signed-rank test as appropriate. The categorical variables were described as frequencies (percentages) and compared by the Chi-squared test or Fisher's exact test as appropriate.

A *p*-value <0.05 was considered statistically significant.

The analysis was performed using R software version 3.6.2 (R Foundation for Statistical Computing, Vienna, Austria).

## Results

### DTA Functional and Safety Parameters Assessment With Different Setups

The data collected on the functional and safety parameters of the DTA connected to the EPT GCB, different Medtronic R&D components, and the Rhythmia^TM^ EAM are detailed in Table 1.

**Table 1:** Functional and safety parameters with different setups.

**Functional and safety parameters observed with setup 0.0**.					
**Load (Ohm)**	**50**			**150**	
**Max power programed on RFG (W)**	**50**	**30**	**15**	**50**	**30**
Difference between the power output indicated on RFG and the power measured at the tip of the DTC with the electrosurgery analyzer during ablation (W)	3	2	1	1	1
**Functional and safety parameters observed with setup 1.1**					
**Load (Ohm)**	**50**			**150**	
**Max power programed on RFG (W)**	**50**	**30**	**15**	**50**	**30**
Difference between the power output indicated on RFG and the power measured at the tip of the DTC with the electrosurgery analyzer during ablation (W)	3	3	1	1	1
**Functional and safety parameters observed with setup 1.2**					
**Load (Ohm)**	**50**			**150**	
**Max power programed on RFG (W)**	**50**	**30**	**15**	**50**	**30**
Difference between the power output indicated on RFG and the power measured at the tip of the DTC with the electrosurgery analyzer during ablation (W)	4	2	1	1	1
Maximum variation in current measured at the tip of the DTC using the electrosurgery analyzer between the 3 measurements performed (mA)	4	0	0	0	0
**Functional and safety parameters observed with setup 3.1**					
**Load (Ohm)**	**50**			**150**	
**Max power programed on RFG (W)**	**50**	**30**	**15**	**50**	**30**
Difference between the power output indicated on RFG and the power measured at the tip of the DTC with the electrosurgery analyzer during ablation (W)	4	3	1	1	1
Maximum variation in the current measured at the tip of the DTC using the electrosurgery analyzer between the 3 measurements performed (mA)	1	0	0	0	0
**Functional and safety parameters observed with setup 3.2**					
**Load (Ohm)**	**50**			**150**	
**Max power programed on RFG (W)**	**50**	**30**	**15**	**50**	**30**
Difference between the power output indicated on RFG and the power measured at the tip of the DTC with the electrosurgery analyzer during ablation (W)	4	3	1	1	1
Maximum variation in the current measured at the tip of the DTC using the electrosurgery analyzer between the 3 measurements performed (mA)	4	0	0	0	0
**Load (Ohm)**	**50**			**150**	
**Functional and safety parameters observed with setup 3.3**					
**Load (Ohm)**	**50**			**150**	
**Max power programed on RFG (W)**	**50**	**30**	**15**	**50**	**30**
Difference between the power output indicated on RFG and the power measured at the tip of the DTC with the electrosurgery analyzer during ablation (W)	4	3	1	1	1
Maximum variation in the current measured at the tip of the DTC using the electrosurgery analyzer between the 3 measurements performed (mA)	4	0	0	0	0
**Load (Ohm)**	**50**			**150**	

*DTC, DiamondTemp catheter; RFG, radio frequency generator*.

At the lowest load setting of 50 Ohm, a maximum discrepancy of 5W could be observed between the maximum power programmed to be delivered by the DTA RFG, the actual power output indicated on the DTA RFG, and the power output measured at the tip of the DTA.

Variations on the current output measured at the tip of the DTA by the RF analyzer could be observed between the three measurements performed with the same settings. All variations were within the limits specified by the manufacturer of the device (6). The results are summarized in Table 1.

### DTA Location Accuracy With Rhythmia^TM^ EAM

The DTA location was represented in real-time on the Rhythmia^TM^ EAM while connected per IFU to the Maestro connection box. Proper tracking of the DTA was observed by visual comparison of the physical catheter movements and its representation on the EAM. This was consistent with all the setups tested.

The accuracy of reference markers displayed on the Rhythmia^TM^ EAM could not be assessed as the Rhythmia^TM^ EAM does not have predefined information on their locations in the wet tank. The baseline points taken using the Intella Map Orion catheter's matrix were tracked using magnetic localization. The verification of the location of baseline points was established by taking points at the reference markers on the wet tank.

Although the catheter was reconnected after each RF energy delivery, no points were taken after the DTC reconnection because it was displayed in the same location it was found prior to its disconnection.

In setup 3.2, no major shifts were observed in the DTC location after RF1, RF2, or RF3 were performed with all setups tested (Table 2). The location of the DTC did not significantly shift in space when compared to baseline reference points. This was consistent after reinsertion and reconnection as well as following RF energy delivery. The distances measured between the baseline points after each variable, for each setup tested, are described in Figures 4, 5 and Tables 2, 3.

**Table 2:** DiamondTemp catheter location after RF1, RF2 or RF3 for all setups tested.

**CONTROL VALUES: baseline points**	**Point 1**	**Point 2**	**Point 3**	**Point 4**	**Point 5**
Distance to baseline points before any other testing variables (mm)	NA	NA	NA	NA	NA
Distance to baseline points after reinsertion of the DTC in the phantom (mm)	NA	NA	NA	NA	NA
Distance to baseline points after reconnection of the DTC (mm)	0.0	0.0	0.0	0.0	0.0
Distance to baseline points after RF1 was performed (mm)	0.9	1.2	1.1	1.1	1.2
Distance to baseline points after reinserting the DTC following RF1 (mm)	0.4	0.6	1.0	1.4	1.2
Distance to baseline points after reconnecting the DTC following RF1 (mm)	0.4	0.6	1.0	1.4	1.2
Distance to baseline points after RF2 was performed (mm)	1.0	1.5	1.6	1.2	1.9
Distance to baseline points after reinserting the DTC following RF2 (mm)	0.5	0.4	1.2	1.6	1.0
Distance to baseline points after reconnecting the DTC following RF2 (mm)	0.5	0.4	1.2	1.6	1.0
Distance to baseline points after RF3 was performed (mm)	0.9	1.2	0.9	1.4	1.2
Distance to baseline points after reinserting the DTC following RF3 (mm)	0.5	0.8	1.0	1.8	1.6
Distance to baseline points after reconnecting the DTC following RF3 (mm)	0.5	0.8	1.0	1.8	1.6
Distance to baseline points after variables tested (mm)	NA	NA	NA	NA	NA
**Quantitative data on Rhythmia MS reliability when connect to the DTA using setup 3.1**					
**Registered baseline points**	**Point 1**	**Point 2**	**Point 3**	**Point 4**	**Point 5**
Distance to baseline points before any other testing variables (mm)	NA	NA	NA	NA	NA
Distance to baseline points after reinsertion of the DTC in the phantom (mm)	NA	NA	NA	NA	NA
Distance to baseline points after reconnection of the DTC (mm)	0.0	0.0	0.0	0.0	0.0
Distance to baseline points after RF1 was performed (mm)	0.5	0.5	1.2	2.5	12.4
Distance to baseline points after reinserting the DTC following RF1 (mm)	1.2	1.5	3.5	4.5	9.8
Distance to baseline points after reconnecting the DTC following RF1 (mm)	1.2	1.5	3.5	4.5	9.8
Distance to baseline points after RF2 was performed (mm)	1.5	1.0	4.0	9.6	10.5
Distance to baseline points after reinserting the DTC following RF2 (mm)	0.4	0.6	1.4	11.8	12.0
Distance to baseline points after reconnecting the DTC following RF2 (mm)	0.4	0.6	1.4	11.8	12.0
Distance to baseline points after RF3 was performed (mm)	2.9	2.8	4.9	10.0	10.2
Distance to baseline points after reinserting the DTC following RF3 (mm)	0.6	0.8	1.0	12.0	12.2
Distance to baseline points after reconnecting the DTC following RF3 (mm)	0.6	0.8	1.0	12.0	12.2
Distance to baseline points after variables tested (mm)	NA	NA	NA	NA	NA
**Quantitative data on Rhythmia MS reliability when connect to the DTA using setup 3.2**					
**Registered baseline points**	**Point 1**	**Point 2**	**Point 3**	**Point 4**	**Point 5**
Distance to baseline points before any other testing variables (mm)	NA	NA	NA	NA	NA
Distance to baseline points after reconnection of the DTC (mm)	0.0	0.0	0.0	0.0	0.0
Distance to baseline points after RF1 was performed (mm)	0.9	1.2	1.1	3.1	3.2
Distance to baseline points after reinserting the DTC following RF1 (mm)	0.4	0.6	1.0	2.4	2.2
Distance to baseline points after reconnecting the DTC following RF1 (mm)	0.4	0.6	1.0	2.4	2.2
Distance to baseline points after RF2 was performed (mm)	1.0	1.5	1.6	3.2	3.9
Distance to baseline points after reinserting the DTC following RF2 (mm)	0.5	0.4	1.2	1.6	2.0
Distance to baseline points after reconnecting the DTC following RF2 (mm)	0.5	0.4	1.2	1.6	2.0
Distance to baseline points after RF3 was performed (mm)	0.9	1.2	0.9	3.4	4.2
Distance to baseline points after reinserting the DTC following RF3 (mm)	0.5	0.8	1.0	1.8	2.6
Distance to baseline points after reconnecting the DTC following RF3 (mm)	0.5	0.8	1.0	1.8	2.6
Distance to baseline points after variables tested (mm)	NA	NA	NA	NA	NA
**Registered baseline points**	**Point 1**	**Point 2**	**Point 3**	**Point 4**	**Point 5**
Distance to baseline points before any other testing variables (mm)	NA	NA	NA	NA	NA
Distance to baseline points after reinsertion of the DTC in the phantom (mm)	NA	NA	NA	NA	NA
Distance to baseline points after reconnection of the DTC (mm)	0.0	0.0	0.0	0.0	0.0
Distance to baseline points after RF1 was performed (mm)	17.2	15.5	21.5	24.5	25.5
Distance to baseline points after reinserting the DTC following RF1 (mm)	10.5	10.4	11.1	12.5	12.4
Distance to baseline points after reconnecting the DTC following RF1 (mm)	10.5	10.4	11.1	12.5	12.4
Distance to baseline points after RF2 was performed (mm)	18.5	21.0	22.0	24.7	32.5
Distance to baseline points after reinserting the DTC following RF2 (mm)	10.9	10.1	11.4	11.5	13.9
Distance to baseline points after reconnecting the DTC following RF2 (mm)	10.9	10.1	11.4	11.5	13.9
Distance to baseline points after RF3 was performed (mm)	20.2	20.7	21.2	25.0	30.0
Distance to baseline points after reinserting the DTC following RF3 (mm)	10.6	11.2	10.9	12.0	12.0
Distance to baseline points after reconnecting the DTC following RF3 (mm)	10.6	11.2	10.9	12.0	12.0
Distance to baseline points after variables tested (mm)	NA	NA	NA	NA	NA

*DTC, DiamondTemp catheter; RF, radio frequency*.

**Table 3:** Distances measured between the baseline points after each variable, for each setup tested.

**Point 1 (*p*-value)**	**Baseline**	**Setup 3.1**	**Setup 3.2**	**Setup 3.3**
**Baseline**	NA	0.99	1.00	<0.001
**Setup 3.1**	0.99	NA	0.99	<0.001
**Setup 3.2**	1.00	0.99	NA	<0.001
**Setup 3.3**	<0.001	<0.001	<0.001	NA
**Point 2 (** ***p*** **-value)**	**Baseline**	**Setup 3.1**	**Setup 3.2**	**Setup 3.3**
**Baseline**	NA	0.99	1.00	<0.001
**Setup 3.1**	0.99	NA	0.99	<0.001
**Setup 3.2**	1.00	0.99	NA	<0.001
**Setup 3.3**	<0.001	<0.001	<0.001	NA
**Point 3 (** ***p*** **-value)**	**Baseline**	**Setup 3.1**	**Setup 3.2**	**Setup 3.3**
**Baseline**	NA	0.93	1.00	<0.001
**Setup 3.1**	0.93	NA	0.93	<0.001
**Setup 3.2**	1.00	0.93	NA	<0.001
**Setup 3.3**	<0.001	<0.001	<0.001	NA
**Point 4 (** ***p*** **-value)**	**Baseline**	**Setup 3.1**	**Setup 3.2**	**Setup 3.3**
**Baseline**	NA	0.002	0.98	<0.001
**Setup 3.1**	0.002	NA	0.93	0.002
**Setup 3.2**	0.98	0.93	NA	<0.001
**Setup 3.3**	<0.001	0.002	<0.001	NA
**Point 5 (** ***p*** **-value)**	**Baseline**	**Setup 3.1**	**Setup 3.2**	**Setup 3.3**
**Baseline**	NA	<0.001	0.98	<0.001
**Setup 3.1**	<0.001	NA	0.003	0.025
**Setup 3.2**	0.98	0.003	NA	<0.001
**Setup 3.3**	<0.001	0.025	<0.001	NA

### Specific Observations Related to the Setup 3.1

In this configuration, a significant shift was observed after the first RF application (Figure 3); however, further applications caused not further shift. This may be linked to the fact that the EPT GCB used for testing was previously extensively used (Tables 2, 3).

### Observations Related to the Setup 3.2

In this configuration, no significant shift has been observed. All points were taken within the Rhythmia catheter location accuracy specifications (1) and were demonstrated to be accurate (Figures 4, 5; Tables 2, 3).

### Specific Observations Related to the Setup 3.3

Setup 3.3 showed a massive shift in catheter position before ablation compared to baseline points acquired using the Orion catheter as a reference. The distance between the baseline points and the points taken after RF1 was over 10 mm. Further delivery of the RF energy caused more significant catheter shift (Tables 2, 3).

## Discussion

The main results of the current study are as follows: (1) a universal method for compatibility assessment of the ablation catheters and navigation systems was described; (2) DTA is compatible with Rhythmia EAM with a safety and reliability profile within the specification; and (3) careful setup is mandatory to achieve good clinical outcomes as only setup 3.2 (Figures 1, 4, 5) demonstrated satisfactory results. Other tested configurations have shown significant accuracy mismatch with reference points taken using a sensor-enabled catheter. The shift was further increased during RF delivery.

The 3D mapping systems have become a standard tool for the diagnosis and treatment of various cardiac arrhythmias. They have multiple benefits like reducing radiation time and dose and improving the precision of ablation treatment (9). However, the use of third-party therapeutic catheters is limited as there is no compatibility out of the box. Extending the range of compatible ablation systems with various 3D mapping platforms allows innovative therapeutic modalities to be used without limitations, which may translate into a clinical benefit.

The *in vitro* compatibility assessment is a crucial step, which must be done prior to the clinical trial. The described method can be a universal solution for such compatibility testing since it is based on industry standards and manufacturer work instructions to evaluate all the parameters. Using only commercially available and CE-marked components during the evaluation makes further clinical use possible.

In the current study, compatibility testing for the DTA and the Rhythmia EAM was performed, assessing all the setups in terms of safety and functionality within specifications. However, in terms of location accuracy, only one setup has shown results within manufacturer specifications and the other 2 configurations were associated with a significant catheter representation shift. This is critical during ablation procedures, and setup 3.2 should be used for further clinical evaluation.

## Conclusions

A universal and reproducible solution for compatibility testing between various mapping systems and ablation catheters has been described. DTA has been demonstrated to be compatible with Rhythmia EAM with satisfactory results if specific setup is used.

## Data Availability Statement

The original contributions presented in the study are included in the article/supplementary material, further inquiries can be directed to the corresponding author/s.

## Ethics Statement

This study was reviewed and approved by Commissie Medische Ethiek, UZ Brussel.

## Author Contributions

Conception and design of the study: LP, IE, and CA. Substantial contributions to the acquisition of data for the study: RR, DC, MV, AG, AS, CM, IO, GB, AA, ES, JS, PB, ML, G-BC, and CA. Substantial contributions to the analysis of data for the study: LP, IE, and RR. Substantial contributions to the interpretation of data for the study and revising the draft of the work critically for important intellectual content: DC, MV, AG, AS, CM, IO, GB, AA, ES, JS, PB, ML, G-BC, and CA. Drafting the study: LP and IE. Final approval of the version to be published and agreement to be accountable for all aspects of the work in ensuring that questions related to the accuracy or integrity of any part of the study are appropriately investigated and resolved: LP, IE, RR, DC, MV, AG, AS, CM, IO, GB, AA, ES, JS, PB, ML, G-BC, and CA. All authors contributed to the article and approved the submitted version.

## Conflict of Interest

IE and DC are employees of Medtronic Inc. MV is employee of Boston Scientific. PB received compensation for teaching purposes from Biotronik. ML is consultant for Atricure. G-BC received compensation for teaching purposes and proctoring from Medtronic, Abbott, Biotronik, Boston Scientific, and Acutus Medical. CA receives research grants on behalf of the center from Biotronik, Medtronic, Abbott, LivaNova, Boston Scientific, AtriCure, Philips, and Acutus. CA received compensation for teaching purposes and proctoring from Medtronic, Abbott, Biotronik, Livanova, Boston Scientific, Atricure, and Acutus Medical Daiichi Sankyo. The remaining authors declare that the research was conducted in the absence of any commercial or financial relationships that could be construed as a potential conflict of interest.

## Publisher's Note

All claims expressed in this article are solely those of the authors and do not necessarily represent those of their affiliated organizations, or those of the publisher, the editors and the reviewers. Any product that may be evaluated in this article, or claim that may be made by its manufacturer, is not guaranteed or endorsed by the publisher.
